# Why did China’s mental health law have a limited effect on decreasing rates of involuntary hospitalization?

**DOI:** 10.1186/s13033-022-00543-w

**Published:** 2022-07-02

**Authors:** Yarong Ma, Jie Zhang, Robert Rosenheck, Hongbo He

**Affiliations:** 1 Affiliated Brain Hospital of Guangzhou Medical University (Guangzhou Huiai Hospital), 36 Mingxin Rd. Liwan District, Guangzhou, 510370 China; 2 Department of Psychiatry, Yale School of Medicine, New Haven, CT USA

**Keywords:** Involuntary hospitalization, Rate, Psychiatric beds, China’s Mental Health Law

## Abstract

**Background:**

China’s Mental Health Law (MHL) implemented in 2013 required increased consideration of the rights of people with mental illness and was expected to lead to a reduction in involuntary hospitalization (IH). This study aimed to examine the rates and correlates of IH in a large psychiatric hospital in Guangzhou from 2014 to 2017 after the implementation of MHL and a structured assessment of the need for IH.

**Methods:**

Unduplicated electronic medical records concerning all inpatients admitted to the hospital with a primary psychiatric diagnose were examined. Diagnostic, sociodemographic and socioeconomic data were used to identify correlates of IH using bivariate chi-square tests followed by logistic regression analysis.

**Results:**

Of 10, 818 hospitalized patients, there was a significant but small increase, from 71.6 to 74.9% in rates of IH in the years after a structured assessment of need for IH was implemented. Logistic regression analysis showed IH was positively associated with being younger, having a local residence, and a diagnosis of bipolar disorder, schizophrenia spectrum disorders or a substance abuse disorder as compared to those diagnosed with major depressive disorder.

**Conclusions:**

IH did not decrease over the first four years after the implementation of China’s MHL and a structured assessment in 2013 perhaps, reflecting the initiation of a systematic assessment of the need for IH and the relatively low number of psychiatric beds in this area.

## Introduction

Involuntary hospitalization (IH) is legally regulated in most countries [1, 2], but only recently became subject to national regulations China in through a national Mental Health Law (MHL) that went into effect on May 1, 2013 [1]. Regulations for involuntary placement [3], Article 30 of the law required that IH would require that patients manifest “self-harm in the immediate past or current risk of self-harm” or demonstrated “behavior that had harmed others or endangered the safety of others in the immediate past or currently exposed a risk to the safety of others” [4]. After the introduction of the new law, significant changes were expected reflecting an anticipated reduction in the rate of IH [5, 6].

Before the law, IH was believed to be common in China and widely thought to be unjustified in many cases, especially in large psychiatric hospitals. A 2002 study found 81.5% of 2333 psychiatric inpatient hospitalizations in 17 Chinese cities were involuntary [7]. Contrary to expectations, after implementation of the MHL in 2013, one study (n = 797) conducted at 16 psychiatric hospitals across China, found 42% of hospitalizations were IHs, 28% were voluntary, and for 30% of admissions IH status was not determinable from the available records [6]. A more recent publication reported a rate of IH of 79.8% from the Shanghai area, rates that had increased after an initial decline [8].

Despite the lower IH rates in western countries [9] as compared to those in China, some reports indicate that IH rates in estren countries have increased since the 1980s, perhaps due to the decreased number of available psychiatric beds following deinstitutionalization [10, 11]. Availability of more numerous psychiatric beds locally appears to be associated with a decreased proportion with IH [12].

By the end of 2014, the number of available psychiatric beds in Guangzhou, the largest city in Southern China was 26.8 per 100,000 population [13], substantially lower than that in most western countries [14, 15], in which, for example, bed availability ranged from 100/100,000 in Australia and Canada to 80 overall in the OECD countries to about 30 in the US, and perhaps increasing the proportion of IHs. This study aimed to investigate the changing rates of IH following the implementation of China’s Mental Health Law in 2013 in a large psychiatric hospital in Guangzhou and to identify clinical and sociodemographic characteristics associated with IH during the first four years after the law was implemented.

## Methods

### Implementation of administrative procedures

To assure compliance with the new law, all patients who were assessed for hospital admission were invited to be voluntarily hospitalized. Those who refused were evaluated with a 12-item checklist of specific suicidal behaviors, other self-harmful activities, as well as recent violent behavior, threats of violent behavior towards other people, or behaviors that might result in destruction of property (e.g., thorough arson or explosives) and thus met criteria for IH outlined in the MHL (Appendix 1). Specific checked items were not documented in available records but after the evaluating clinician had determined that IH was warranted by the legal criteria, familial agreement was sought (which was also required) and written informed consent was obtained from the patients’ family or legal guardians.

### Study design and participants

The sample for this retrospective observational study was drawn from the electronic medical records of the Affiliated Brain Hospital of Guangzhou Medical University, which operates 1920 beds and hospitalizes approximately 7000 patient annually and constitutes the largest inpatient psychiatric provider in southern China, serving the needs of a catchment area of over a hundred million of inhabitants. Discharge abstracts, documented, demographic information, health insurance coverage, diagnoses, length of stay, numbers of previous hospitalizations, and whether hospitalization was voluntary or involuntary and were derived from the hospital’s electronic medical record system covering hospitalizations from April 1, 2014, to June 30, 2017.

Patients were included in the sample if they had a principal International Classification of Diseases, tenth edition (ICD-10) diagnosis of a psychiatric disorder as defined below. We excluded the diagnosis of mental disorders due to known physiological conditions (F01-F09) (n = 1555), as these patients usually received medical services from the neurology department. Patients with mental retardation (n = 320), childhood mental illnesses (n = 290) or other psychiatric symptoms undiagnosed (n = 267), were also excluded, as the involuntary placement process of the MHL was not typically recommended for these patients. The study protocol was approved by the Affiliated Brain Hospital of Guangzhou Medical University Ethics Committee.

### Measures

Demographic and clinical characteristics included age, sex, marital status, current employment, local residence (i.e., within the city of Guangzhou), health insurance, length of stay (LOS), number of previous hospitalizations, and the ICD-10 diagnosis at discharge. Diagnoses were classified into five subgroups: (1) schizophrenia spectrum disorders (ICD-10 codes F200-259); (2) affective disorders (F300-339) sub-classified as major depressive disorder and bipolar disorders; (3) mental and behavioral disorders due to psychoactive substance use (F10-F19), and (4) anxiety, dissociative, stress-related, somatoform and other nonpsychotic mental disorders (F40-F48). The hospitalization circumstances (voluntary vs. involuntary) was represented by a binary variable assessed during the admission process based on the checklist process described above.

### Statistical analysis

The analysis proceeded in 2 stages. First, the proportion of IHs by an indicator of the year of admission was evaluated using chi-square tests.

Next comparison of involuntary and voluntarily hospitalized patients was conducted using chi square analyses for dichotomous categorical variables while continuous variables were categorized by quartiles. Given the exploratory nature of our study, the significant differences for each test were established at p < 0.05, 2-tailed.

Next, binary logistic regression analysis with forwarding stepwise selection was used to identify demographic and clinical correlates independently associated with IH. Variables that were significant at p < 0.05 were entered into the regression model to calculate adjusted odds ratios (OR) and the sensitivity and specificity of a high-risk categorization in model 1. The association of IH and LOS was examined in a separate second model. Statistical analyses were performed with IBM SPSS Statistics for Windows (Version 25.0. Armonk, NY: IBM Corp.)

## Results

A total of 10,818 patients were included, of whom 72.8 percent (n = 7872) were involuntarily hospitalized. From the year 2014 to 2017, the proportion admitted as IH increased slightly from 2066 of 2884 (71.6%) in 2014; to 2596 of 3623 (71.7%) in 2015; 2283 of 3074 (74.3%) in 2016; and 927 of 1237 (74.9%) in 2017 (chi square = 10.568, p = 0.014).

The comparison of patients who were voluntarily hospitalized (VH group) and involuntarily (IH group) hospitalized showed that there was no significant difference between the groups in either employment or numbers of previous hospitalizations. Patients in the IH group were more likely to be female (chi square = 6.626, p = 0.010), younger (chi square = 108.649, p < 0.001), unmarried (single, divorced, or widowed) (chi square = 41.181, p < 0.001), had a local residence (chi square = 4.575, p = 0.032), greater likelihood of having health insurance covered (chi square = 11.698, p = 0.001), and also had longer lengths of stays (chi square = 446.007, p < 0.001) (Table 1).

**Table 1:** Comparison of patients between voluntary (VH) and involuntary hospitalization (IH)

n (%)	Overall N = 10,818^b^	VH group 2946 (27.2)	IH group 7872 (72.8)	IH rates (%)	χ^2^	*p*
Gender, female	4975 (44.3)	1365 (46.3)	3430 (43.6)	71.5	6.626	0.010
Age, years^a^					108.649	0.000
< 25		711 (24.1)	1955 (24.8)	73.3		
25–34		611 (20.7)	2021 (25.7)	76.8		
34–47		643 (21.8)	2024 (25.7)	75.9		
≥ 47		981 (33.3)	1872 (23.8)	65.6		
Married^c^	4952 (45.8)	1496 (50.8)	3456 (43.9)	69.8	41.181	0.000
Local residence	4977 (46.0)	1306 (44.3)	3671 (46.6)	73.8	4.575	0.032
Employed	3208 (30.0)	853 (29.3)	2355 (30.3)	73.4	0.994	0.319
Insurance covered	4235 (39.1)	1076 (36.5)	3159 (40.1)	74.6	11.698	0.001
Length of stays, days^a^					446.007	0.000
< 17		837 (28.4)	1142 (14.5)	57.7		
17–32		740 (25.1)	1414 (18.0)	65.6		
32–56		787 (26.7)	2491 (31.6)	76.0		
≥ 56		582 (19.8)	2825 (35.9)	82.9		
Previous hospitalizations					5.121	0.163
1	8109 (75.0)	2248 (76.3)	5861 (74.5)	72.3		
2	1730 (16.0)	441 (15.0)	1289 (16.4)	74.5		
3	556 (5.1)	139 (4.7)	417 (5.3)	75.0		
4 or more	423 (3.9)	118 (4.0)	305 (3.9)	72.1		
Diagnosis group^d^					624.091	0.000
Major depressive disorder	1375 (12.7)	597 (20.3)	778 (9.9)	56.6		
SCZ	4460 (41.2)	1042 (35.4)	3418 (43.4)	76.6		
Bipolar disorder	3768 (34.0)	750 (25.5)	2890 (36.9)	76.7		
Substance-related disorder	908 (8.4)	288 (9.8)	620 (7.9)	68.3		
Stress and somatic disorders	405 (3.7)	269 (9.1)	136 (1.7)	33.6		
The year at admission					10.568	0.014
2014	2884 (26.7)	818 (27.8)	2066 (26.2)	71.6		
2015	3623 (33.5)	1027 (34.9)	2596 (33.0)	71.7		
2016	3074 (28.4)	791 (26.8)	2283 (29.0)	74.3		
2017	1237 (11.4)	310 (10.5)	927 (11.8)	74.9		

^a^Categorized by quartiles: age (37.49 ± 15.08 year-old), Length of stays (47.53 ± 68.94 days)

^b^System-missing value: marriage, n = 3(< 0.01%); Employed, n = 119 (1.1%)

^c^Unmarried: single (n = 5133), divorced (n = 571), widowed (n = 159)

^d^*SCZ* Schizophrenia spectrum disorders; Substance-related disorder (F10-F19 in ICD-10); Stress and somatic disorders (F40-F48 in ICD-10)

Patients with bipolar disorder were most likely to be involuntarily hospitalized (2920 of 3768, 76.7%), followed by those with schizophrenia spectrum disorders (3418 of 4460, 76.6%), major depressive disorder (778 of 1375, 56.6%), substance-related disorder (F10-F19) (620 of 908, 68.3%) (chi square = 624.091, p < 0.001), and anxiety, dissociative, stress-related, somatoform and other nonpsychotic mental disorders (F40-F48) (136 of 405, 33.6%) (Table 1).

Forward stepwise logistic regression analysis of IH showed that having a local residence (Odds Ratio, OR = 1.138, 95% CI (confidence interval) [1.008, 1.285], p = 0.037), medical insurance coverage (OR = 1.136, 95% CI [1.001, 1.289], p < 0.001) and being younger were most strongly and significantly associated with IH. Compared with the patients with major depressive disorder, the adjusted odds ratio for IH was significantly greater for those with schizophrenia spectrum disorders (OR = 2.396, 95% CI [2.106, 2.725], p < 0.001), bipolar disorders (OR = 2.784, 95% CI [2.430, 3.190], p < 0.001), or substance-related disorder (OR = 1.597, 95% CI [1.334, 1.911], p < 0.001). Net of these factors IH was significantly associated with a longer length of stays (Table 2).

**Table 2:** Forward stepwise logistic regression for involuntary hospitalization

	B	S.E	Wald	Sig	OR	95% CI	
Model 1							
Local residence = 1	0.129	0.062	4.359	0.037	1.138	1.008	1.285
Age, y (ref. = ” > = 47″)			114.709	0.000			
< 25	0.487	0.066	53.864	0.000	1.627	1.429	1.853
25–34	0.618	0.066	88.987	0.000	1.855	1.632	2.109
34–47	0.537	0.063	72.149	0.000	1.710	1.511	1.935
Insurance covered = 1	0.128	0.064	3.916	0.048	1.136	1.001	1.289
Diagnosis (ref. = MDD)			501.502	0.000			
SCZ	0.874	0.066	176.274	0.000	2.396	2.106	2.725
Bipolar disorders	1.024	0.069	217.465	0.000	2.784	2.430	3.190
Substance-related disorder	0.468	0.092	26.122	0.000	1.597	1.334	1.911
Stress and somatic disorders	− 0.997	0.121	67.817	0.000	0.369	0.291	0.468
Year at admission (ref. = 2014)			10.373	0.016			
2015	0.020	0.058	0.116	0.733	1.020	.911	1.142
2016	0.149	0.061	6.035	0.014	1.161	1.031	1.308
2017	0.183	0.081	5.056	0.025	1.201	1.024	1.408
Model 2^a^							
LOS, days (ref. = ”< 17″)			303.271	0.000			
17–32	0.293	0.067	19.255	0.000	1.341	1.176	1.529
32–56	0.705	0.064	121.093	0.000	2.024	1.785	2.295
≥ 56	1.108	0.068	264.827	0.000	3.029	2.650	3.461

*MDD*  major depressive disorder, *SCZ* schizophrenia spectrum disorders, *LOS* length of stays, *CI* confidence interval

^a^Adjusted for variables in Model 1

## Discussion

In the current study, the overall rates of IH were high at 72.8 percent over all four years and, unexpectedly, increased slightly but significantly from 71.6% in 2014 to 74.9% in 2017. This finding was consistent with a national study of IH in China in which showed 81.5% of 2333 inpatients were involuntarily hospitalized at 17 psychiatric hospitals in 2002 [7]. Similarly, a recent publication from the Shanghai Mental Health Center reported a 79.8% IH rate, and noted that the proportion of IHs increased from 69.7% in May 2013 to 87.8% in May 2015 [8]. Thus, China’s MHL does not seem to have decreased rates of the IH, and several studies, like this one, showed increased rather than decreased rates after implementation of the national mental health law. The continuing high rate of IH may reflect the relatively low numbers of psychiatric bed per 100,000 population in China, as noted in the introduction, and the slightly increased proportion may reflect application of systematic criteria to evaluate the need for IH after implementation of the law.

IH rates remain generally lower in western countries as compared to China, for example, 26% in Ontario, Canada [16], 30.1% in Switzerland [2], 22.4% across five European countries considered together (Belgium, Germany, Italy, Poland, and the UK) [9]. However, a greater than 60% increase in rates of IH was recently observed in the UK, and increase that was closely associated with the decreased provision of psychiatric beds from 166.1 to 63.2 per 100,000 adults between 1988 and 2008 [11], substantially fewer psychiatric beds than that in the leading countries of the European Union, e.g., France (87 beds per 100,000 population) and Germany (127 beds per 100,000 population) [17]. Bed numbers are even lower in the US with 22 beds per 100,000 population [17], in which increasing number of people with serious mental illness have been incarcerated, perhaps viewed as an alternative and unanticipated form of IH [10].

China has far fewer psychiatric hospital beds (31.5 per 100,000 population in 2015) [18] most Western other countries and thus patients who present for evaluation may be more likely to have an urgent need for hospitalization, and to be so severely impaired that their recognition of their need for hospitalization is seriously impaired [16, 19, 20]. Guo et al. [21] estimated that China has a need for as many as 85.6 psychiatric beds per 100,000 population which might sharply reduce the proportion of patients who require IH, by providing the capacity to provide voluntary hospitalization to less severely impaired patients [10].

One further issue of relevance may be the strongly stigmatized attitudes towards mental illness in China [22], making patients more reluctant to acknowledge the severity of their problems. Mohamed et al. compared inpatients diagnosed with schizophrenia in the US and China on measures of insight into illness and acceptance of medication. They found that, after controlling for overall schizophrenia symptom severity, patients in the Chinese sample had far lower scores than the US sample on measures of insight into illness as well as acceptance of their need for medication [23]. They concluded that greater stigma in China led to poorer insight and reluctance to acknowledge illness. This cultural tendency may play an important role in necessitating IH for some patients.

A major limitation of this and other studies from China is that involuntary hospitalization was not systematically documented before the reform law took effect in 2013 and as a result data on baseline rates of IH from before the law went into effect are not available. In addition, detailed responses to checklists were not documented and this study thus had to assume both that the check list was used and that it was used as intended. Furthermore, the sample was based on data from a single medical center, and its generalizability to other hospitals in China is unknown, although our findings clearly fit into a pattern of greater rates of IH in specialized psychiatric hospitals in China after the MH law was implemented.

## Conclusions

In spite of the above limitations, this study found that the proportion of IHs at a large Chinese tertiary psychiatric hospital is substantial and unexpectedly increased after implementation of the national mental health law. The legislation seems to have failed to decrease IH due to the limited number of available inpatient beds in China as well as culturally shaped stigma and resultant patient denial of mental illness.

## Data Availability

The data that support the findings of this study are available from the corresponding author upon reasonable request.

## Appendix 1

See Table

**Table 3:** Checklist for involuntary hospitalizations (translated by Chinese)

During the current episode, patients with one of the situations below should be involuntarily hospitalized
In the case of involuntary I (danger to oneself)
1. With imperative auditory hallucinations that threaten their safety 2. With negative ideas or suicide plans 3. Behaviors to harm themselves already happened 4. With attempted suicide 5. Unable to take care of himself/herself 6. Patients with mental retardation in women with hyper-sexuality and imprudent behavior 7. Abnormal diet may threaten one’s life 8. Stupor or dementia, or more than moderate mental retardation, inability to protect themselves Besides above, when the patient is at risk of endangering his or her own safety, please describe in detail ()
In the case of involuntary II (danger to the others)
1. Verbal threat to another person or in action 2. Declare or yelling to threat as assaulting or retaliating against another person 3. Behaviors of beating and smashing 4. That threats such as arson or explosion do not fall under any of the above circumstances Besides above, when the patient’s condition is at risk of endangering the safety of others, please describe in detail ()

3.

## Abbreviations

IH: Involuntary hospitalization
VH: Voluntary hospitalization
MHL: Mental health law
ICD-10: International Classification of Diseases, tenth edition
LOS: Length of stay
OR: Odds ratio
CI: Confidence interval
